# Exploring and Mitigating Insecticide Resistance in Urban Pests with New Synergists and Insecticides

**DOI:** 10.3390/insects17090970

**Published:** 2026-09-20

**Authors:** Ayashaa Ahmad, Vartul Panhalkar, Manas Sarkar, Jeffrey Bloomquist

**Affiliations:** 1 Department of Research and Development, Vestacy (E.H. Entomology India Pvt. Ltd.), D-30, Infocity II, Sector 33, Gurgaon 122001, India; ayashaa.ahmad@reckitt.com (A.A.); manas.sarkar@reckitt.com (M.S.); 2 Germ Protection—Bar Soap Delivery, Research & Development, Reckitt Benckiser India Pvt. Ltd., Infocity II, Sector 33, Gurgaon 122001, India; vartul.panhalkar@reckitt.com; 3 Emerging Pathogens Institute, Entomology and Nematology Department, University of Florida, Gainesville, FL 32610, USA

**Keywords:** bed bug, cockroach, housefly, insecticide, mosquito, synergist

## Abstract

This review summarizes the impact of insecticide resistance in urban pests and its implications for ‘One Health’ systems. Mosquitoes are the major vector of human disease via blood feeding, followed by houseflies and cockroaches via physical contact, and finally bed bugs, ants, and termites, which are not typically disease vectors. Besides overt disease, other impacts in the context of One Health include nuisance biting and resultant infections, as well as allergic reactions. Control of urban pests is still primarily achieved with insecticides, which have continually improved over time. However, their use has selected for the evolution of resistance to several compound classes across broad geographical areas and multiple species. Resistance results from several types of mechanisms: altered inherited behavioral repertoires that reduce chemical exposure, modified target sites, increased metabolism, and reduced penetration, which have recently been shown to have several specific underlying causes. Overall, the literature portrays a complex resistance landscape that highlights the importance of comprehensive, multifaceted approaches to maintain effective control. New technologies and approaches for resistance management are also discussed, with emphasis on improved chemical control measures via novel synergists and insecticides from both natural and synthetic sources.

## Simple Summary

Urban pest control provides great benefits to human societies through improved hygiene, prevention of vector-borne diseases, and reduced structural damage to buildings. The continued evolution of chemical control to safer and more selective compounds, as well as more targeted applications, has improved pest control efficiency while reducing environmental contamination and exposure to non-target species. However, selection for resistance in urban pest populations threatens the effectiveness of several chemical compound classes across broad geographical areas and especially for major pest species, such as mosquitoes, houseflies, cockroaches, and bed bugs. In this review, we find that the literature on insecticide resistance mechanisms in urban pests portrays a complex landscape that highlights the importance of using a broad range of collaborative efforts to combat these challenges effectively. New technologies and approaches for integrated resistance management in response to these issues are also discussed, with emphasis on improved chemical control measures.

## 1. Introduction and Scope

The focus of this review is on resistance mechanisms and their circumvention in urban pests in the context of One Health, which is a collaborative, multidisciplinary approach encompassing local, regional, national, and global levels to optimize health outcomes by recognizing the interconnectedness of human, animal, and plant populations, and the environment [1]. The review is broad in scope but will emphasize specific examples of general principles in lieu of exhaustive literature coverage. Resistance to major chemical classes of insecticides will be discussed across several taxonomic groups of major pests, including cockroaches, houseflies, bed bugs, and mosquitoes. Although these pests are cosmopolitan and not strictly urban, perhaps their greatest impact is on urban areas, and they represent pest species for which the authors have experience and expertise. Topics related to mosquitoes are included where appropriate for coverage but are not an overarching emphasis, given the large number of research articles and reviews published in recent years on mosquito resistance as it impacts vector control. The limited information on resistance in social insect pests, such as ants and termites, will also be briefly mentioned, as it may increase insecticide usage and off-target effects. Other human parasites (e.g., lice, mites, ticks, and fleas), as well as lawn and garden pest control, which are also important in urban ecosystems, will not be covered. Approaches to circumvent resistance will be mostly limited to discussion of novel synergists and insecticides.

For the purposes of this review, literature searches on insecticide resistance included scientific databases such as PubMed, ScienceDirect, Web of Science, and Google Scholar. Keywords and phrases such as “insecticide resistance,” “mosquitoes,” “cockroaches,” “mechanisms,” “pesticide resistance,” etc., were used alone or in various combinations to access relevant studies and articles. The selection process emphasized peer-reviewed research and relevant review articles on mechanisms contributing to insecticide resistance in urban species, as well as reports of new synergists and insecticides. Quality and relevance assessment of selected articles involved several criteria defined as follows. The rigor of the experimental design was evaluated, including whether the studies were conducted in controlled environments or in the field, and the statistical methods used. We considered articles published in reputable journals or information from reliable websites, with citations and impact factors used to evaluate quality. Further, whether the results of a study have been generally confirmed by other investigators was assessed for possible generalization of the findings.

## 2. One Health Impacts from Urban Pests and Insecticide Usage

One Health impacts of urban pests can occur through a number of different aspects. The vectorial capacity of mosquitoes is undoubtedly the major concern, which is under intense investigation, and need not be documented here. However, other urban pests can also serve as vectors, especially filth flies and cockroaches. A review of studies documenting the carrying of human pathogens by houseflies (*Musca domestica*) found that they harbored >130 human pathogens, predominantly bacteria [2]. It was concluded that the evidence for transmission of these pathogens is circumstantial, although there is a correlation between the incidence of human diarrhea and housefly populations [2]. Similarly, cockroaches can be a reservoir and vector for a diverse range of pathogens, including bacteria, viruses, fungi, and parasites found on their cuticle or in their digestive tract [3]. German cockroaches (*Blattella germanica*), in particular, are also known to elicit human allergic reactions to shed cockroach proteins [4]. Bed bugs (*Cimex* spp.) can carry blood-borne pathogens but are not known to be effective vectors of disease [5]. Instead, the primary medical impact of bed bugs arises from allergic reactions to salivary components resulting in dermal inflammation. Likewise, the exoskeleton of household ants (*Monomorium* spp.) can harbor potentially pathogenic bacteria, and in one study the bacterial species count was 75% greater in indoor ant samples as opposed to those taken outdoors [6]. Although it can be envisioned that termite infestations could also cause respiratory or allergic problems in buildings, we did not find peer-reviewed documentation of this effect.

## 3. Insecticide Resistance in the Urban Environment

Numerous insecticides are used by homeowners and pest control professionals in houses, restaurants, hospitals, hotels, etc., in urban environments, and various factors impact resistance development and human health. Several principal insecticide chemical classes, such as organophosphates (OPs), pyrethroids, neonicotinoids, and insect growth regulators (IGRs), are registered for use in urban pest management [7]. However, repeated and indiscriminate use of insecticides leads to resistance and higher use levels, including by homeowners themselves [8], thereby posing increased risk to both human and animal health and chemical load in the environment, as well as complicating resistance management efforts [8].

Resistance development can be influenced by multiple factors, such as the frequency and type of insecticide use, species involved, resistance mechanism and intensity, genetic inheritance, initial frequency, as well as local insecticide usage patterns and environmental conditions. Moreover, resistance distribution is fluid and can vary by geographic location, and variations in resistance mechanisms have been observed, highlighting the importance of understanding local contexts in managing resistance. Some regions have high levels of resistance, while others do not, and populations in close geographical locations tend to be more similar than those at greater distances, according to the First Law of Geography [9]. Thus, another important caveat is to acknowledge that resistance documented and explored in the laboratory may not directly reflect control failure in the field.

In a global context, the study by Tantely et al. [10] evaluated Culex spp. mosquitoes on La Réunion, an isolated island east of Madagascar in the Indian Ocean. Resistance was prominent to all tested insecticides, including organochlorines, organophosphates, and pyrethroids. The isolated nature of the island would tend to preclude natural in-migration of resistant alleles, suggesting they were selected locally. Otherwise, the worldwide shipping of goods and human movement can be responsible for the translocation of urban pests that are not strong fliers, including cockroaches and bed bugs.

Multiple urban pest species have developed resistant populations [11,12,13,14,15]. It is well known that control of mosquito-borne diseases has become problematic due to the development of resistance, particularly to pyrethroids. The housefly (*Musca domestica*) is known to be resistant to 62 unique insecticides [15], including organochlorines, organophosphates, carbamates, and pyrethroids. Although resistance in houseflies is often documented in animal production facilities, it can have relevance for urban pest control as well. Cockroaches are considered the most common pests in urban buildings, as well as restaurants, schools, etc. The German cockroach in particular has shown various levels of resistance to neonicotinoids (imidacloprid), oxadiazines (indoxacarb), and the phenylpyrazole fipronil, as well as high levels of resistance to pyrethroids [16,17]. In the past two decades, a worldwide resurgence in the bed bugs *Cimex lectularius* (L.) and *Cimex hemipterus* (F.) has been observed, and bed bug control has centered on pyrethroids, and pyrethroid resistance in bed bug populations has exploded [18]. Thus, insecticide resistance has greatly impacted bed bug management, and domiciliary eradication has become challenging and expensive [19].

Compelling data on insecticide resistance are still relatively scarce for social insects; however, some reduced insecticide sensitivity has been documented [20]. Dark rover ants (*Brachymyrmex patagonicus* Mayr) and white-footed ants (*Technomyrmex difficilis* Forel) showed significant species-wide tolerance to the insecticide fipronil [21,22]. A survey by Brill and Buczkowski [23] examined the susceptibility of the odorous house ant (*Tapinoma sessile* [Say]) to three insecticides: fipronil, lambda-cyhalothrin, and dinotefuran, all commonly used in ant control. The study found that dinotefuran sensitivity was lower in urban colonies relative to colonies from other locales and that queens were significantly more tolerant compared to workers across all insecticides tested. Several studies have reported significantly reduced insecticide efficacy against Formosan subterranean termite (*Coptotermes formosanus* Shiraki) for soil-applied termiticides from the chlorinated hydrocarbon, pyrethroid, organophosphate, carbamate, and phenylpyrazole classes, as reviewed by Scharf and Lee [20]. Similarly, there is a report that sand termite strains from Egypt showed pyrethroid resistance of >100-fold, depending on the specific strain and compound under study [24].

## 4. Mechanisms of Insecticide Resistance in Urban Pests

A better understanding of insecticide mode of action and the mechanisms by which insects become resistant is crucial for effective pest control through the design of integrated resistance management (IRM) strategies [25]. IRM can be addressed by the development of more targeted and effective pesticides, thereby ensuring efficient management of pest populations. This approach also involves designing methods to overcome insecticide resistance, such as the use of synergists to block metabolic resistance enzymes, or rotating modes of action and using mixtures of active ingredients to retard resistance selection. Understanding resistance mechanisms will provide the information necessary to increase sustainable insect management by enabling more precisely directed pest control methods. The result will be less chemical use, thereby reducing collateral exposure to beneficial insects, wildlife, and humans and facilitating the use of safer and more efficacious alternatives. Additionally, it will improve existing integrated pest management strategies and promote long-term solutions rather than short-term fixes [26].

Insecticide resistance mechanisms present in houseflies, mosquitoes, bed bugs, and cockroaches have been the subject of extensive research over the past several decades, which has revealed multiple mechanisms that contribute to overall resistance [27,28]. In general, molecular genetic PCR analysis for detecting resistance alleles, especially for monitoring target site and metabolic mechanisms, is more common than subsequent functional studies to characterize them. Discussion of resistance mechanisms in urban pests is given below, with a summary given in Table 1. Original findings are given to indicate scientific publication priority, along with previous reviews to summarize well-established findings, as well as more recent studies.

### 4.1. Behavioral Resistance

As a first line of defense, an organism can develop strategies to reduce its exposure to a lethal dose of a pesticide by genetically inherited changes in behavior [27,28]. When more specifically defined, as reviewed by Hubbard and Murillo [29], behavioral resistance in general can be categorized as either stimulus-dependent or stimulus-independent. Behavioral resistance that is stimulus-dependent relies upon the increased ability to detect and limit contact with a toxic substance before acquiring a lethal dose, usually due to a repellent or irritant property of the compound or its formulation, including how its presentation is perceived. Behavioral resistance that is stimulus-independent reflects behavior that leads to avoidance of an environment where insecticide exposure might occur, likely from innate behavioral patterns and pathways. Overall, there are relatively fewer studies of behavioral resistance compared to physiological and biochemical mechanisms. Possible reasons include the lack of standard behavioral assays, or it might be the necessary complications of behavioral analysis [27]. This can be especially significant if the resistance is polygenic in nature, as in the case of bait avoidance [28], as presented below.

#### 4.1.1. Excito-Repellency

One of the first documented forms of behavioral resistance, excito-repellency, was first observed in houseflies and mosquitoes, and then bed bugs and other species [29]. Avoidance of DDT residues by mosquitoes and houseflies could not be attributed to physiological resistance, and further behavioral analysis found that the insects quickly left treated areas, which prevented them from picking up a lethal dose. Excito-repellency can have two components: the contact irritancy of an insecticide or a non-contact spatial repellency [29]. Further, this spatial repellency could result from olfactory detection of a chemical or a more non-specific hyperkinesis. More detailed analysis of the mechanisms involved is beyond the scope of this review.

#### 4.1.2. Mosquito Exophily

Behavioral resistance can also result from selection of populations having a different behavioral repertoire from that typically observed. A classic example of this behavioral adaptation (stimulus-independent) is increased exophily of *Anopheles gambiae* mosquitoes [30]. This resistance is expressed as preferred outdoor feeding behavior in areas where indoor usage of long-lasting insecticidal nets (LLIN) removed individuals that rested on interior walls after taking a blood meal [30]. The resistance is not due to enhanced excito-repellency but instead results from a population shift in behavior. Additional examples of this behavioral resistance mechanism are available from a review by Gatton et al. [31], where a failure to reduce malarial parasite rates (*Anopheles* sp.) by indoor residual spraying (IRS) in Northern Nigeria was because of outdoor resting behavior after a blood meal. This behavioral change was also observed in *An. funestus* females in Tanzania [31].

#### 4.1.3. Bait Avoidance

Bait avoidance is a common type of resistance in urban pests, and pertinent examples include housefly and German cockroach populations. In cockroaches, aversion to the active ingredient chlorpyrifos was a major factor underlying reduced bait efficacy, with a much smaller contribution from physiological mechanisms [32]. Glucose aversion (GA) in German cockroaches is a well-defined mechanism resulting from changed gustatory perception of glucose from sweet to bitter [33]. Thus, the continuous use of glucose-containing baits triggered behavioral resistance by altering gustation and therefore reducing bait consumption and insecticide exposure [34]. GA also impacts the residual killing of nymphs by coprophagy as well as the reproductive success of female German cockroaches. Behaviorally significant levels of glucose are present in adult feces upon which nymphs feed, but GA nymphs avoid this fecal food source and the active ingredient also present [35]. The sugar fondness of female cockroaches is exploited by male roaches who release secretions containing maltose and related sugars to attract the females. However, a GA-resistant female senses these secretions as a deterrent, thus impacting sexual and reproductive behavior, which in turn has induced compensatory adjustments in the sugar blend of the nuptial secretions to restore mating success [35].

Behavioral resistance in the housefly has been observed in choice or no-choice bioassays with malathion-, methomyl-, and azamethiphos-containing baits, the latter due to inert bait components as reviewed by Hubbard and Murillo [29]. Resistance to imidacloprid-containing baits has also been observed in the housefly, with the following characteristics. The resistance was polygenic, incompletely recessive or dominant, and resulted in >90% survival of both male and female houseflies with no increase in physiological resistance, as indicated by complete susceptibility in no-choice tests. In addition, the resistance was remarkably specific, extending only to imidacloprid and not to six other neonicotinoids in parallel experiments [29]. Additional information on the mechanisms involved remains to be collected.

#### 4.1.4. Particle Covering of Treated Surfaces

A recent study by Wen et al. [36] reported behavioral resistance in the invasive fire ant (*Solenopsis invicta*), where the insects deposited soil particles on top of treated surfaces to avoid contact. These authors showed that *S. invicta* covered surfaces treated with fipronil, rotenone, or avermectin with more soil particles compared to control surfaces, and this effect was dose-dependent. Alternatively, no increase in particle movement was observed with beta-cypermethrin, a known fire ant repellent, indicating that this resistance is likely impacted by multiple mechanisms. Wang and Henderson [37] suggested this type of behavioral resistance may be underreported, since most ant repellent studies are laboratory-based, where particles are not available for the colonies, or in fields where the observations were limited to a relatively short duration, e.g., less than 1 h.

### 4.2. Target Site Resistance

Target site resistance is a reduction in the expression level or in the ability of an insecticide to bind to and modify the function of a target [38]. The three groups of insecticide targets that express this form of resistance are enzymes, ion channels, and neurotransmitter receptors.

#### 4.2.1. Modified Acetylcholinesterase (MACE)

Acetylcholinesterase (AChE) is the target for organophosphorus (OP) and carbamate insecticides, and modified AChE (MACE) occurs through various, often shared mutations of the *ace* gene encoding AChE and, depending on the species and mutation, can favor resistance to OPs and/or carbamates [39]. In *Anopheles* and *Culex* mosquitoes, major mutations in AChE have identified the G119S, F290V, and F331W substitutions [39,40]. In contrast, mutations involving the ace gene of *Aedes aegypti* have been less well documented [41], perhaps because more than one mutation event is required to change glycine to serine at codon 119 in *Ae. aegypti*, which is a major resistance mutation in mosquitoes [42]. *Musca domestica* shows five different types of mutations in *ace*, including V260L, A316S, G342A, G342V and F407Y [39]. A genotypic study of *ace* alleles in 12 different laboratory strains and two field-collected strains of housefly [43] found 15 alleles associated with organophosphate resistance. Liming et al. [44] identified three mutations (V261L, G343A, and F408Y) located near the enzyme active site that gave inhibition constant (ki) ratios > 20 for carbamates, but lower ratios for OPs. The AChE mutation F348Y is found in bed bugs and contributes to both OP and carbamate resistance [45]. Initial studies to document resistant AChE in four German cockroach strains found no differences in inhibitor sensitivity and concluded that MACE was not common in this species [46]. Another analysis of 14 strains of *B. germanica* from the USA, Panama, Denmark, and Dubai found that only the strain from Dubai had less sensitive AChE [47]. A recent review of resistance mechanisms in German cockroaches [48] confirms there still remains a paucity of peer-reviewed information on specific AChE mutations conferring resistance in this species.

#### 4.2.2. Knockdown Resistance (kdr) in the Voltage-Sensitive Sodium Channel (VSSC)

The VSSC is responsible for action potential generation, and knockdown resistance (kdr), encompassing mutation(s) in the VSSC, is the major mechanism of target site resistance and modifies sodium channel sensitivity to these compounds [49]. Co-occurrence of multiple *kdr* mutations has been commonly associated with higher levels of phenotypic resistance to DDT and pyrethroids. Williamson et al. [50] identified the specific mutations associated with kdr by sequence analysis of housefly strains carrying *kdr* (L1013F) and the more resistant *super-kdr* factor (M918T) and provided a plausible mechanism that explained why the latter is especially effective in providing protection against pyrethroids having an alpha-cyano-3-phenoxybenzyl alcohol. Bed bugs have been shown to carry six kdr-associated mutation sites (Y/L995H, V1010L, I1011F, L1014F, V1016E, L1017F/S) related to pyrethroid resistance [51]. In German cockroaches, the L993F mutation in kdr is the most prevalent, and when coupled with additional mutations E434K and C764R, results in higher levels of resistance [52,53]. Multiple mutations providing increased levels of resistance are also observed for homologous sequences in mosquitoes [12]. Finally, mutational and *Xenopus* oocyte expression analysis of the kdr gene from *Ae. aegypti* found evidence for five mutations comprising two putative pyrethroid binding sites that reduced Na^+^ channel sensitivity [54].

#### 4.2.3. Resistance to Dieldrin (rdl) in the γ-Aminobutyric Acid (GABA) Receptor

The molecular basis of cyclodiene resistance at the GABA receptor-chloride channel complex was initially found in *Drosophila melanogaster* as an A302S mutation in the *rdl* subunit [55]. Gao et al. [56] searched for the A302S mutation in thousands of houseflies sampled from the U.S. in 2007 but found no evidence for it. Subsequently, it was shown by Ozoe et al. [57] that a cyclodiene-resistant OCR strain of housefly possesses an A299S mutation in the GABA receptor that confers the expected resistance to channel-blocking compounds. There is evidence that reduced target site sensitivity contributes to fipronil resistance in German cockroaches and is due to the A302S mutation [58,59]. Although the A302S mutation has been identified in *Ae. albopictus* mosquitoes [60], other studies identified a homologous A296G substitution in *rdl* of *An. gambiae* [61], which is also linked to resistance in *An. funestus* in concert with the additional mutation V327I [62]. In oocyte expression studies, *An. gambiae* rdl receptors carrying the A296G mutation were 14-fold less sensitive to fipronil inhibition in vitro [63]. In contrast, molecular screening of nine different strains of bed bugs for this mutation has so far only observed the susceptible isoform [64]. Although the A302S mutation has not been identified in houseflies or bed bugs, it may appear in the future, and other mutations may be present.

#### 4.2.4. Nicotinic Acetylcholine Receptor (nAChR)-Based Resistance

The nicotinic acetylcholine receptor (nAChR), located primarily at the postsynaptic membrane [65], is the target site for neonicotinoid insecticides, and it is responsible for mediating excitatory cholinergic neurotransmission in the central nervous system of invertebrates [66]. Persistent activation of this receptor by neonicotinoids such as clothianidin causes repetitive nerve firing and membrane depolarization [67]. Although target site resistance mechanisms in this receptor have been well documented in agricultural pests, they have so far been rarely encountered in urban pest species. It has been shown that reduced expression of the Mda2 subunit of the housefly nAChR correlates with reduced toxicity of neonicotinoids [68]. A recent study of field-collected *An. gambiae* identified increased expression frequencies of mutations in Agβ1 and Agα6 that correlated with acetamiprid and clothianidin resistance [69], but functional analysis linking these mutations to reduced nAchR sensitivity to insecticides was not performed.

### 4.3. Metabolic Resistance

Metabolic resistance occurs via elevated enzyme activities that lead to increased degradation or sequestration (binding) of insecticides, thereby preventing them from reaching the target site. Metabolic detoxification is typically associated with the major enzyme systems discussed below.

#### 4.3.1. Carboxylesterases (CBE)

CBE metabolism is a common mechanism of resistance in many insects to organophosphate, carbamate, and pyrethroid insecticides, hydrolyzing ester bonds to produce the corresponding alcohols and acids as metabolites [70]. Besides hydrolysis, sequestration from high-affinity binding of insecticides is also a possible resistance mechanism of CBEs [71]. A mutation in the housefly *MdαE7* gene (G137D) has been associated with diazinon resistance [72] and multiple mutations in the same gene with spinosad resistance [73]. Increased CBE production is the primary factor associated with resistance and occurs via two genetic mechanisms: gene amplification and gene regulation [74]. Two genes, *estα2* (esterase alpha 2) and *estβ2* (esterase beta 2), are involved in CBE amplification and overexpression in *Culex quinquefasciatus* [75]. CBEs are associated with fipronil resistance and degrade pyrethroids in urban pests. According to Sarkar et al. [76], esterase production, when exposed to fipronil, was greater in a resistant strain of the mosquito *C. quinquefasciatus* than in a susceptible strain. Increased activity of CBE was detected in beta-cypermethrin-resistant *M. domestica* when compared to susceptible [77]. Numerous additional studies have demonstrated increased expression levels of CBE after insecticide selection in the lab [78] and/or elevated catalytic activity in cockroach field strains [47]. Finally, Lee [79] has reviewed the resistance status and mechanisms in *Cimex* spp., and CBE enzymes have been implicated in this group as well.

#### 4.3.2. Cytochrome P450 Monooxygenases (CYP)

Insect cytochrome P450s are hemoproteins that can oxidize widely diverse substrates and produce a vast array of monooxygenase reactions resulting in more polar and less toxic metabolites [80]. Overexpression of P450 genes occurs by gene amplification or by cis- or trans-acting regulators in resistant populations/strains [80]. In the housefly, elevated expression of multiple P450 genes is responsible for pyrethroid resistance, including *CYP6D1* in the LPR strain, as well as several other allelic forms in this species [81]. Vontas et al. [82] reported differential expression of genes in one susceptible and two resistant strains of *An. gambiae* mosquitoes. They found increased transcription of peptidases, sodium/calcium exchangers, and genes implicated in lipid and carbohydrate metabolism, as well as the cytochrome P450 gene *CYP314A1* in the DDT-resistant ZAN/U strain, highlighting the complexity of an organism’s response to insecticide selection. Increased expression of mRNA levels for one cytochrome P450 gene (*CYP397A1V2*) in a pyrethroid-resistant bed bug was suggested to underlie resistance to a wide variety of insecticides, such as DDT, deltamethrin, permethrin, and imidacloprid [83]. Similarly, Gaire et al. [84] found that a field-collected Knoxville strain of bed bug was 290,000-fold resistant to topical application of deltamethrin at the LD_50_ level, attributed to cytochrome P450 activity by piperonyl butoxide (PBO) synergism. Overexpression of P450s is also observed in cockroaches [81]. For example, in German cockroaches from California, *CYP6K1* was significantly overexpressed, 2.1–5.8 fold greater, in all field-collected strains [85].

#### 4.3.3. Glutathione-S-Transferase (GST)

GST is another primary detoxifying enzyme system in urban pests, and GST-mediated resistance results from the chemical addition of the tripeptide glutathione to insecticides at halogen or alkyl (especially methyl) positions, leading to the production of less toxic and more water-soluble conjugates [86]. GSTs have been organized into six classes: theta, sigma, zeta, omega, delta, and epsilon [87]. In DDT- and permethrin-resistant *Ae. aegypti*, GSTe2 of the epsilon class is overexpressed in all life stages, and the increases ranged from 1.4-fold in pupae to 2.9-fold in adult males [88]. *GSTe5* was overexpressed to a maximum of 16.9-fold in adult females, and *GSTe7* was 7.8-fold greater in the resistant strains. In *An. gambiae*, epsilon-class GSTs (in particular *agGSTe2*) are also associated with resistance to DDT and are overexpressed in resistant individuals [89]. In the housefly, resistance-conferring GSTs underlie resistance to DDT and diazinon [90], and two epsilon-class GSTs (*MdGST6A* and *MdGST6B*) are believed to be involved in insecticide resistance [91]. Several strains of the bed bug *C. lectularius* (L.) showed enhanced activity of GSTs compared to a susceptible strain [92]. Besides a role in insecticide resistance [48], allergies to German cockroaches arise, at least in part, from Bla g epitopes (e.g., Bla g 5) resulting from shed *B. germanica* GSTs [4], with similar antigenicity of GSTs from *Periplaneta americana* [93].

### 4.4. Penetration Resistance

Penetration resistance serves to retard the uptake of xenobiotics into the insect and is known to be involved in cross-resistance to multiple insecticides, usually acting in concert with other mechanisms as a multiplier. This type of resistance has historically been poorly understood, but recent research has revealed a surprising number of underlying mechanisms. Both physical and metabolic processes can contribute to penetration resistance, and in some cases, the mechanisms overlap, where enhanced metabolic capacity results in altered cuticular lipids, as reviewed below. Further, the extent of resistance can vary by compound. A recent study found that a resistant strain of housefly showed 7.1-fold penetration resistance to fluralaner, but less toward five other insecticides, and the mechanism remains unknown [94].

#### 4.4.1. Altered Cuticle Structure

Thickened cuticles have been documented as a resistance mechanism in houseflies, cockroaches, bed bugs, and mosquitoes. Thickening of the insect cuticle reduces the rate of contact toxicant uptake and consequently delays access to target sites and provides more time for detoxifying enzymes to work [95]. In the German cockroach, cuticular protein BgCPLCP1 was recently shown to contribute to insecticide penetration resistance [96]. Silencing *BgCPLCP1* by RNAi resulted in ca. 86% and 82% thinner cuticle in insecticide-susceptible and β-cypermethrin-resistant strains, respectively. Concomitantly, the thinner and more permeable cuticles after RNAi exposure reduced survival by 14.4% and 20.0% in β-cypermethrin-treated susceptible and resistant strains, respectively [96]. In a highly pyrethroid-resistant strain of bed bug, morphometric analysis revealed a significantly (*p* < 0.001) thicker cuticle than that of a susceptible strain [97]. Similarly, in *Ae. aegypti* larvae of a deltamethrin-resistant strain had a cuticle nearly twice as thick as that of susceptibles [98]. In adult *An. funestus* tarsal cuticle thickness was significantly increased in pyrethroid-resistant individuals compared to susceptibles [99], and a similar cuticular thickening, especially of the procuticle, was found in tarsal segments of pyrethroid-resistant *An. gambiae* [100].

#### 4.4.2. Altered Cuticular Lipids

Altered cuticular hydrocarbon (CHC) amounts and composition are related to reduced insecticide uptake and resistance, whether or not in the presence of cuticle thickening. A study by Chen et al. [101] confirms that CYP4G19 monooxygenase, which is involved in hydrocarbon production in *B. germanica,* is overexpressed in a resistant strain and causes ca. 3-fold penetration resistance to beta-cypermethrin. Likewise, Balabanidou et al. [100] have shown significantly increased CHC production in resistant *An. gambiae*, especially straight-chain alkanes (n-C30, n-C28, and n-C29), a monounsaturated straight-chain alkene (n-C31: 1), as well as a monomethyl branched alkane (3-methyl C30), and two dimethyl branched alkanes (dimethyl C39 and dimethyl C41). Moreover, these changes were more pronounced in leg cuticles than in cuticles from the main mosquito body [100].

#### 4.4.3. Increased Cuticle Epithelial Transporters

Transporter proteins in the cuticular epithelium serve to extrude CHC for cuticle maintenance and also transport of insecticides and in the latter case slowing penetration to target tissues [102]. More specifically, Kefi et al. [103] found that the ABC transporter ABCH2 was highly expressed in the legs of *Anopheles coluzzii*. RNAi-mediated silencing of *ABCH2* increased the knockdown of deltamethrin from 23% to 90% and mortality from 55% to almost 99%. Penetration studies using [^14^C]deltamethrin showed increased penetration of radiolabeled insecticide in constructs of *An. coluzzii* lacking the transporter after a short 4 min contact with a 0.01% treated paper. Similarly, immunofluorescence analysis of *Culex pipiens pallens* found that ABCG6032427 transporter expression was highest in the legs, and RNAi knockdown of this transporter increased sensitivity to deltamethrin [104]. Scanning and transmission electron microscopy in this study also showed reduced cuticular thickness in this species, along with loss of morphological integrity. In the bed bug *C. lectularis*, four ABC transporter genes, *Abc8*, *Abc9*, *Abc10*, and *Abc11,* showed increased expression in a deltamethrin-resistant strain [105]. We have been unable to identify any published work that specifically implicates ABC transport of xenobiotics underlying penetration resistance in cockroaches or houseflies, and we would note their conspicuous absence as well in the recent review of this topic by Amezian et al. [102].

#### 4.4.4. Upregulation of Insecticide Sequestering Proteins

Pu and Chung [106] have reviewed an emerging resistance mechanism that affects overall penetration and distribution of insecticides. Reduced bioavailability from this mechanism is distinct from the previously described sequestration by metabolic enzymes and results from binding sequestration by upregulation of leg chemoreceptor-associated proteins. These proteins possess a hydrophobic pocket and include sensory associated proteins (SAP) that transport hydrophobic materials through the sensillum lymph and are highly overexpressed in the appendages of resistant insects, especially SAP2. In mosquitoes, RNAi silencing of *SAP2* reduced resistance to deltamethrin, permethrin, and alpha-cypermethrin in the highly resistant Tiassale strain of *An. gambiae* without any change in mortality to other insecticide classes [107]. The prevalence of this mechanism in other urban pest species remains to be documented.

**Table 1:** Summary examples of resistance mechanisms in urban pest species, with references and abbreviations as provided in the text.

Mechanism	Sub-Category	Pest Group and Citation(s)
Behavioral	Excito-repellency	Mosquitoes [29]
	Exophily	Mosquitoes [30,31]
	Bait avoidance	Houseflies [29] Cockroaches [32,33,34,35]
	Covering treated surfaces	Ants [36,37]
	MACE	Mosquito [39,40,41,42] Housefly [39,43,44] Bed bug [45], Cockroach [47]
Target site	kdr	Mosquito [12,49,54] Housefly [50], Bed bug [51] Cockroach [52,53]
	rdl	Housefly [57], Cockroach [59,60], Mosquito [61,62,63,64] Bed bug [65]
	nAChR	Housefly [69], Mosquito [70]
	CBE	Mosquito [72,75,76,77] Housefly [73,78] Cockroach [47,79], Bed bug [80]
Metabolism	P450	Mosquito [80,82], Housefly [80,81]. Bed bug [83,84], Cockroach [81,85]
	GST	Mosquito [86,88,89] Housefly [90,91] Cockroach [48], Bed bug [92]
	Cuticle structure	Cockroaches [96], Bed bugs [97] Mosquito [98,99,100]
	Cuticular lipid composition	Mosquito [100], Cockroach [101]
Penetration	Epithelial transporters	Mosquitoes [102,103,104] Bed bugs [105]
	Sequestering proteins	Mosquito [107]

## 5. Resistance Intervention via Novel Synergists and Insecticides

While analyses of single treatments vs. mixtures, etc., are useful for evaluating resistance interventions in the field and other methods are available, we restrict discussion to select examples of new synergists and insecticides (Table 2). Going forward, more investigation into new synergists should have utility, noting that synergism occurring in one species might not universally apply to others. The future will also likely bring the introduction of novel insecticidal materials to the urban environment, recognizing that historically many compounds used for urban pest control were re-purposed from agricultural applications.

### 5.1. Commercial and Diagnostic Synergists That Inhibit Metabolism

**Figure 1:**
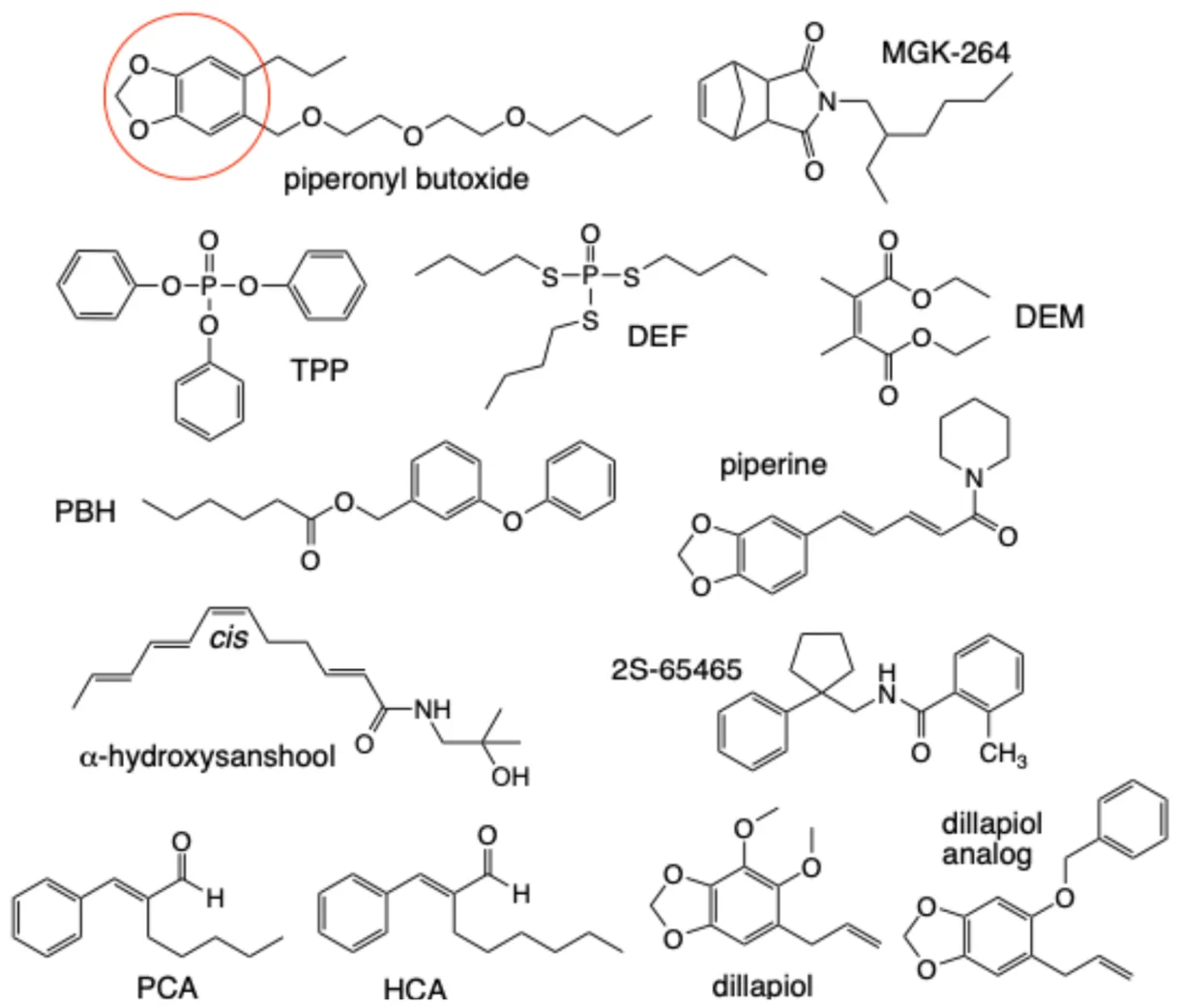
Commercial and experimental synergists with abbreviations as given in the text. The methylenedioxyphenyl moiety of PBO, the ‘warhead’ (red circle) that reacts with P450 monooxygenases, is present in dillapiol and its analogs, as well as piperine.

Synergists are natural or synthetic chemicals, used at nontoxic amounts, that are known to increase the lethality and effectiveness of insecticides [108]. They have been used commercially for over 80 years. Synergists typically inhibit the metabolic enzymes that break down insecticides, thereby increasing toxicity, and a wide variety of compounds have been evaluated for synergistic activity [108]. The classical commercial synergists (Table 2; Figure 1) registered for use in urban pest control are PBO and *N*-octylbicycloheptenedicarboxamide (MGK-264), both of which are cytochrome P450 inhibitors [109].

A classical use of synergists in resistance diagnostics is given in Gonzalez-Morales and Romero [110], who studied PBO, as well as diethyl maleate (DEM, an inhibitor of GSTs), and *S*,*S*,*S*-tributylphosphorotrithioate (DEF) and triphenylphosphate (TPP), which are inhibitors of CBEs (Table 2; Figure 1). The impact of these materials was studied on deltamethrin efficacy in two pyrethroid-resistant *C*. *lectularius* L. strains. Significant synergist ratios (SR) were recorded in a highly resistant field-derived strain with a resistance ratio (RR) of 20,000. The four synergists were used with deltamethrin (PBO SR = 20.5; DEM SR = 11.7; DEF SR = 102.3; and TPP SR = 9.7). In a less resistant strain of bed bug (RR = 3333), significant synergism was observed with pretreatment of PBO and DEM (PBO SR = 158.8; DEM = 58.8); DEF and TPP had no synergistic effect. The data provide examples of detoxification enzymes playing significant and differential roles in bed bug resistance to deltamethrin. Although CBE and GST inhibitors have proven useful for identifying the contribution of these enzymes to resistance in numerous studies, neither of these synergist classes has been commercially registered, and they remain an unexploited tool of resistance management. An alternative approach for leveraging these metabolic systems for insecticide synergism would be to use dsRNAi to reduce their expression, an approach under consideration for agricultural pests [111].

### 5.2. Experimental Chemical Synergists

Given the proven utility of synergists for increasing activity of insecticides and combating resistance, future efforts should be made to develop new synergist molecules, provided regulatory hurdles can be overcome. Concerns include increased toxicity to mammals and honeybees in addition to the target pest. More recent results suggest that target site effects may contribute to synergism, besides inhibition of metabolism. Specific examples are given below.

Hardstone et al. [112] synthesized 3-phenoxybenzyl hexanoate (PBH) as a multi-functional pyrethroid synergist (Table 2; Figure 1), acting as a surrogate substrate for both CBEs (the hexanoate ester) and P450 monooxygenases (3-phenoxybenzyl moiety). Addition of PBH to permethrin-treated females of a resistant strain (ISOP450) of *Culex pipiens quinquefasciatus* caused a 3-fold increase in toxicity. Similarly, with deltamethrin, the synergism was 6-fold in females of *C*. *lectularius* (SR = 6.2), and in compound synergized toxicity of permethrin (8.6-fold) and deltamethrin (7.2-fold) against adult *Ae. aegypti* mosquitoes, compared to 3- to 4-fold for PBO, respectively. The synergism was not due to reduced metabolism or enhanced penetration, but instead via augmentation of pyrethroid effects on the nerve membrane [113]. It has not been tested on other urban pests.

### 5.3. Natural Product Synergists

A large number of recent studies, reviewed by Isman and Norris [114], have evaluated essential oils or their constituents as bioinsecticides and synergists for mosquitoes and other urban pests. Here, we will emphasize their role as synergists, since many essential oils synergize conventional chemical insecticides, including pyrethroids and neonicotinoids, among others, against mosquitoes, houseflies, bed bugs, and cockroaches (Table 2). For example, Gaire et al. [84] have shown that blockage of P450 metabolism by essential oil constituents enhances deltamethrin toxicity in a pyrethroid-resistant strain of bed bug. However, it should be noted that in some cases, a neutral effect or antagonism of toxicity has also been observed [115], indicating specificity of action and that the facile assumption of positive synergism by essential oils should be avoided.

The mechanisms of synergism by essential oils can vary and have more than one mechanism, including but not limited to metabolic inhibition. Increased penetration of insecticide by co-application of essential oils is well documented [114]. Moreover, increased penetration can be inferred by an increase in knockdown, but a recent study with fir needle oil found that this effect may not correlate with increased 24 h toxicity, probably due to delayed induction of P450 activity [116]. It is also known that enhancement of insecticide effects on the nervous system by essential oils can occur, but this mechanism remains mostly unexplored and is not well defined [115]. Recent studies on dillapiol and its analogs (Table 2; Figure 1) showed that they synergized the toxicity of pyrethrum to Colorado potato beetle via P450 inhibition and/or GST inhibition [117]. While these studies were carried out on agricultural pests, the fact that they strongly synergized pyrethrum suggests that they would be useful for synergizing pyrethrum on urban pest species as well. Natural product alkamides isolated from black pepper (*Piper nigrum*), such as piperine (Table 2; Figure 1), have long been recognized as having synergistic effects, which can fall under different mechanisms. Piperine has low insecticidal activity but can act as an effective insecticide synergist, which occurs via inhibition of cytochrome P450 enzymes and efflux transporters [118]. A recent study has shown that both black pepper extract containing alkamides and Sechuan pepper extract containing alpha-hydroxysanshool (Table 2; Figure 1) can synergize the toxicity of natural pyrethrins against adult mosquitoes [119] and also synergize the nerve block by pyrethrins on the mosquito larval central nervous system. Another pepper extract, Cha Plu from *Piper sarmentosum* (Table 2; Figure 1), has been found to be a strong synergist of natural pyrethrins (SR = 10) and clothianidin (SR = 24) against female *Aedes aegypti* [120] and could synergize the blocking effect of natural pyrethrins on the mosquito larval central nervous system at treatments as low as 2.5 ng/mL (ppb). The relative contributions of individual Cha Plu constituents to this potent synergism remain to be identified, as well as its effects on other urban pests.

A novel proposal for synergist deployment has been put forward by Liu et al. [121], who evaluated compounds with high binding affinity to insecticide-sequestering chemosensory proteins in *An. gambiae*. The hypothesis is that displacing insecticides from these binding proteins would increase the amount of free insecticide and augment toxicity. They identified two analogs of the natural product cinnamaldehyde, alpha-pentylcinnamaldehyde (PCA) and alpha-hexylcinnamaldehyde (HCA) (Table 2; Figure 1). These compounds increased the toxicity of deltamethrin to fourth-instar *An. gambiae* larvae augmented with binding protein; thus, the synergism presumably acted by this mechanism. Further support for this mechanism came from molecular docking and molecular dynamics computer simulations, as well as isothermal titration calorimetry analysis of competitive binding with pyrethroids and site-directed mutagenesis of AgSAP1 [121].

### 5.4. Novel Experimental Insecticides

**Figure 2:**
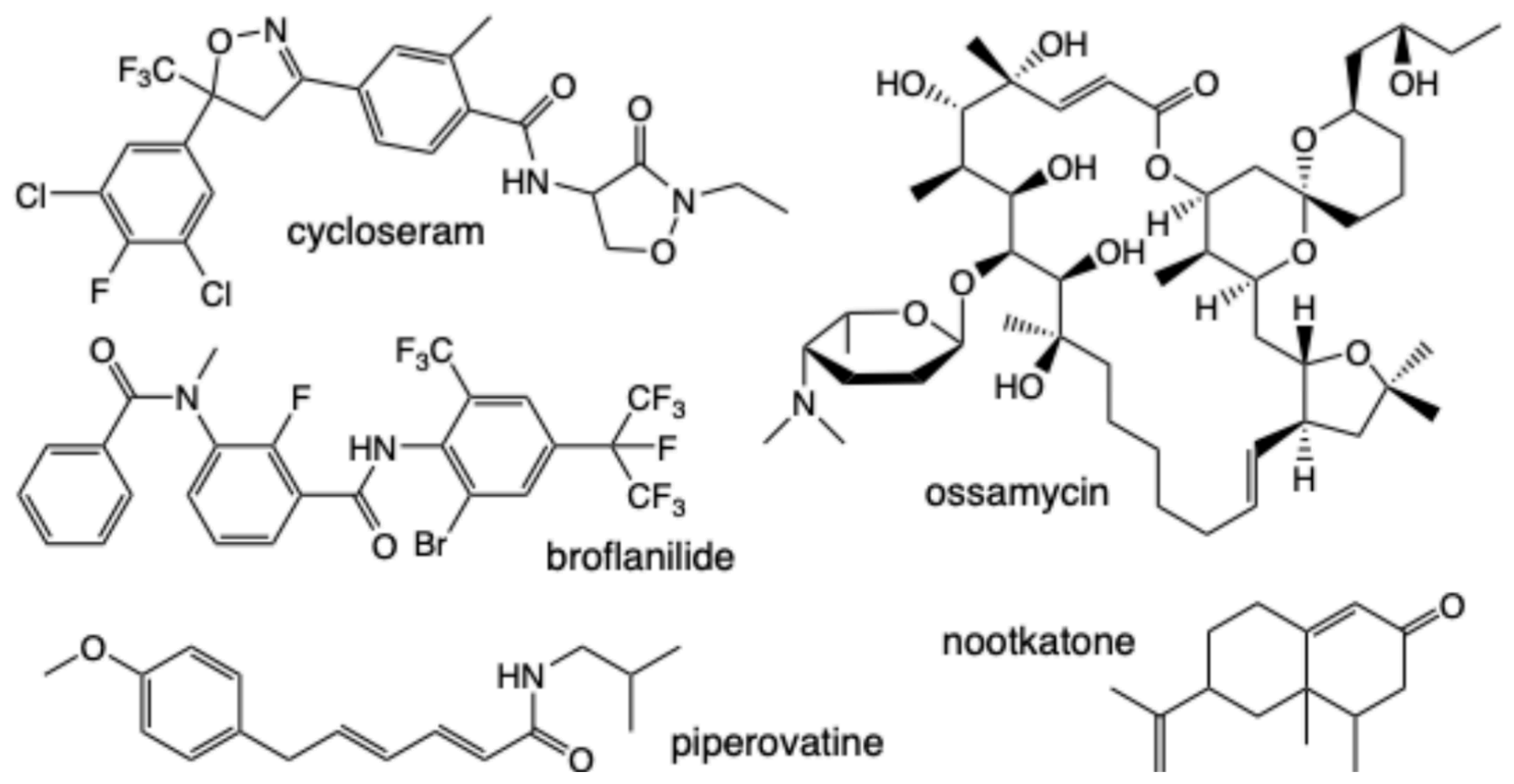
Chemical structures of novel insecticidal molecules evaluated against urban pests or their associated targets. The chiral centers of ossamycin are shown to illustrate its chemical complexity.

New insecticidal molecules will continue to be repurposed for urban pest control, as seen over time with many compounds, including pyrethroids, fipronil, indoxacarb, and spinosyns. Numerous studies have evaluated natural products as insecticides (biopesticides), with pepper alkamides showing promise. These molecules are known to be insecticidal via an action on voltage-sensitive sodium channels and are less affected by kdr resistance [122]. Evaluation of piperovatine (Table 2; Figure 2) found that its insecticidal activity was also expressed via open-state modification of the *B. germanica* sodium channel in oocyte expression assays [123]. Note that it lacks the methylenedioxyphenyl moiety of the P450-inhibiting alkamide piperine, dillapiol, and PBO (Figure 1). Similarly, the natural product nootkatone (Table 2; Figure 2) is an EPA-registered repellent that is also toxic to a number of arthropod species via GABA receptor antagonism with an interesting rdl resistance profile [124]. This compound showed no cross-resistance in a strain of *An. gambiae* carrying the *rdl* mutation A302G [125] but was rendered less active by the A302S mutation found in *Drosophila melanogaster* [124]. This differential resistance is presumably due to the smaller side chain of A302G permitting the binding of nootkatone [124].

A study by Engdahl et al. [126] serves as an additional example of insecticidal natural products toxic to urban pests. They screened numerous compounds from natural sources against mosquito larvae and found that among the most active was ossamycin (Table 2; Figure 2), isolated from *Streptomyces hygroscopicus* var. *ossamyceticus*. In 72 h, it was 100% lethal to larvae at 0.5 µM, and other studies have shown it acts via mitochondrial ATPase inhibition [127]. Its complicated structure as a macrocyclic lactone with multiple chiral centers is reminiscent of the avermectins and requires purification from bacterial isolates, as has been accomplished with the avermectins in an economical fashion. Finally, the newer GABA antagonists broflanilide, a diamide [128], and the isoxazoline cycloseram [129] have been evaluated for activity against urban pest species (Table 2; Figure 2). The latter compound was recently shown to be toxic at levels comparable to commercial products in both treated surface and topical bioassays to cockroaches, ants, termites, and bed bugs. It is expected that the new materials presented here would initially be unaffected by most existing resistance mechanisms in the field.

**Table 2:** Examples of resistance intervention tools via natural and synthetic synergists and insecticides, along with representative references.

Intervention	Examples	Citations
Commercial Synergists	PBO, MGK-264	[109]
Diagnostic Synergists	DEF, TPP, DEM	[108,109,110]
Novel Synthetic Synergists	PBH 2S-65465	[112,113]
Natural Product	essential oils	[84,114,115,116]
Synergists	dillapiol and analogs	[117]
	pepper extracts and components	piperine [118] Sechuan [119] Cha Plu [120]
	cinnamaldehydes	[121]
Novel Insecticidal Molecules	natural products	piperovatine [123] nootkatone [124] ossamycin [126]
	synthetics	broflanilide [128] cycloseram [129]

## 6. Conclusions, Data Gaps, and Future Studies

There have been a large number of studies on resistance mechanisms and their use as diagnostic tools in urban pests. These insects have evolved an elaborate array of different mechanisms to allow them to avoid or survive insecticide exposure. The distribution of resistant alleles differs across the pests covered here. Some mechanisms, such as enhanced metabolism by P450, CBE, and GST enzymes, as well as MACE and kdr, are ubiquitous (Table 1). Others, such as nAchR, are expressed rarely, so far only in houseflies and mosquitoes, and characterization of this mechanism is incomplete. Recently, it has been shown that penetration resistance occurs through several different molecular mechanisms across these pests, but so far none of them are uniformly present. IRM program implementation further depends on dissemination and archiving of data generated through research and field surveillance of these mechanisms, which remains a challenge, along with data integrity requirements for different control programs/situations, as exemplified by the efforts of the IRAC.

Despite the large number of studies on urban pest resistance mechanisms, the management of resistance and decision-making for urban pests remains problematic. Resistance management with respect to crop protection is different from urban insect/vector control management. As mentioned previously, urban pest control generally inherits repurposed agrochemical insecticides for use in urban biomes. This situation results in a more limited number of available insecticidal actives and possible combination treatments. Other factors slowing the introduction of new insecticides for urban pest control can include operational factors, such as allowable cost for low-income areas. This situation results in a paucity of actives with different modes of action, which are required for effective resistance management. This situation is exemplified by mosquito control, where there are limited insecticide choices outside of pyrethroids, especially on LLINs, and resistance to these molecules is widespread, demonstrating an acute need for new insecticides. It is encouraging that the new GABA antagonists broflanilide and cycloseram are or will soon be deployed, and they should not be affected by rdl resistance.

Moreover, urban pest control only has commercially registered synergists, PBO and MK-264, as P450 inhibitors. Thus, if regulatory hurdles can be overcome, new synergists (e.g., PBH) that block multiple enzyme systems could have a great positive impact. Natural products could also be a source of new synergist molecules, and essential oils or their components could play an expedited role here, either as synergists or new insecticidal formulations in their own right. Finally, to secure future success, continued research and collaborative efforts on these topics are required, continually shaped by advancements in technology and sustainability to hold down costs, reduce nontarget exposure, and minimize ecological impact.

## Data Availability

No new data were created or analyzed in this study. Data sharing is not applicable to this article.
